# Association of major dietary patterns with socioeconomic status, obesity, and contracting COVID-19 among Iranian adults

**DOI:** 10.3389/fnut.2024.1301634

**Published:** 2024-01-29

**Authors:** Maryam Maharat, Mehran Rahimlou, Ali Sioofi, Seyedeh Forough Sajjadi, Seyedeh Parisa Moosavian

**Affiliations:** ^1^Department of Community Nutrition, Vice-Chancellery for Health, Shiraz University of Medical Sciences, Shiraz, Iran; ^2^Department of Nutrition, School of Public Health, Zanjan University of Medical Sciences, Zanjan, Iran; ^3^Student Research Committee, Shiraz University of Medical Sciences, Shiraz, Iran

**Keywords:** dietary pattern, weight, COVID-19, socioeconomic status, adults

## Abstract

**Background:**

The coronavirus disease 2019 (COVID-19) pandemic clearly affected the lifestyle and dietary habits of millions of people worldwide. The purpose of this study was to evaluate the association of major dietary patterns with socioeconomic status, obesity, and contracting COVID-19.

**Methods:**

We conducted a cross-sectional study using an online survey for data collection with a total of 1,187 participants (over the age of 18 years) who reported their sociodemographic details, anthropometric index (weight and height), and dietary intake. Multivariable logistic regression models were applied to assess the association between major dietary patterns and study outcomes.

**Results:**

A total of 1,106 adults were included in the current analysis. We identified three major dietary patterns (plant-based, meat, and Western dietary patterns). The mean age of participants was significantly higher in the upper tertile of plant-based dietary patterns (DPs) compared to the first tertile, while it was lower in the top tertile of meat and Western DPs. The percentage of participants who lived in urban areas was significantly higher in the third tertile of meat DP than in the first tertile (*p* < 0.001). Moderate adherence to Western DP was significantly associated with increased odds of obesity (OR: 1.79; 95% CI: 1.17, 2.74). In addition, high adherence to Western DP was significantly related to increased odds of obesity after controlling for confounders. Subjects in the second tertile of the Western DP had higher odds (95% Cl: 1.04, 1.92) for COVID-19 infection than the first tertile.

**Conclusion:**

This study showed that moderate and high adherence to a Western dietary pattern was associated with a higher risk of obesity and COVID-19 infection during the pandemic. Future studies are needed to confirm these findings.

## 1 Introduction

Coronavirus disease-2019 (COVID-19), caused by a severe acute respiratory syndrome coronavirus 2 (SARS-CoV-2), has become a global public health crisis of this century (1). The pandemic situation and the measures needed to control the spread of the disease have resulted in lockdowns and many restrictions on daily living, with probable consequences on lifestyle, particularly affecting physical activity, sleep quality, eating habits, and mental health (2–4). Available evidence has shown several unhealthy lifestyles during the COVID-19 pandemic, including increased sedentary behavior and less time spent on physical exercise (5).

Moreover, negative emotions (e.g., stress and boredom), sleep disorders, and sedentary behaviors caused by home confinement could promote overconsumption, change dietary patterns, and reduce diet quality (6). These changes result in higher consumption of “comfort foods” and energy imbalance. These foods, mainly rich in simple carbohydrates, unhealthy fat, and sugar, are associated with an increased risk for more severe complications of COVID-19 (7, 8), thereby increasing the risk of developing non-communicable diseases (NCDs), such as obesity, diabetes, and cardiovascular disease (9).

On the other hand, obese subjects are one of the groups with a higher risk for COVID-19 complications (10). Kompaniyets et al. (11) reported that obesity is associated with a 3.07 times higher risk of hospitalization and a 1.42 times higher risk of severe illness when hospitalized. Obesity-induced adipose tissue expansion contributes to increased circulating levels of proinflammatory cytokines (12). This low-grade inflammation state could further exacerbate the inflammation in patients with COVID-19 (13).

Xu et al. observed that prudent dietary patterns, which are characterized by high intake of fresh fruits and vegetables, and low intake of soft drinks and fried food were negatively associated with weight gain among the Chinese population during the COVID-19 pandemic (13). In addition, a healthy dietary pattern, which is based on healthy fats, plant food (fruits, vegetables, cereals, and legumes), and low-fat protein foods, could support the immune system in fighting infections such as COVID-19 (14, 15). A recent meta-analysis showed that the effectiveness of a plant-based diet against COVID-19 infection was 50% (16). Moreover, an observational study indicated that unhealthy or traditional dietary patterns were associated with a higher risk of severe COVID-19 (17).

Dietary patterns are mainly shaped by income and food prices. A decrease in the financial capacity to purchase food due to job instability during the pandemic would lead to adjusting the items of food baskets with more affordable resources (18).

Therefore, the current study is conducted to determine the association of major dietary patterns with socioeconomic status, obesity, and contracting COVID-19 among Iranian adults.

## 2 Methods and materials

### 2.1 Study design and population

The present cross-sectional web-based survey was performed among Iranian adults during the COVID-19 pandemic.

The required sample size was calculated using Gpower software (α = 0.05, β = 0.2) according to a previous study. Therefore, the estimated sample size for the study is 515 volunteers. A combination of convenience and snowball sampling was used to employ participants.

Participants aged 18 years or above and living in Iran were considered as inclusion criteria. Participants excluded from the study were those (1) who were pregnant or lactating, (2) who were on specific diets, and (3) who did not fill out the questionnaire appropriately or reported invalid data.

### 2.2 Study variables and tools

An online questionnaire with an invitation letter was posted through different social media portals (including Instagram and WhatsApp). To prevent duplicate responses, we used an online form that allows only one response per user. We also asked the participants to share the study link to increase the number of persons who receive the invitation link. The purpose of the survey was provided on the cover page of the online questionnaire to inform the participants.

An online questionnaire was prepared in which participants self-reported sections regarding categories of sociodemographic characteristics, food consumption, and anthropometric index (weight and height) during the COVID-19 pandemic.

Dietary intake was assessed using a modified food frequency questionnaire (FFQ). The validity and reliability of the FFQ were evaluated previously (18). The correlation coefficient for reliability was 0.77. Since this survey was conducted during the COVID-19 pandemic, we summarized the questionnaire to prevent the adverse effects of the length of the questionnaire on the response rate. Each item included a typical portion size. Participants reported each food consumption based on servings per week.

Self-reported data on weight and height were used to calculate BMI by dividing weight (kg) by high squared (m2) and interpreted according to the criteria of the World Health Organization. Following this, the participants were classified into groups: (i) underweight (BMI < 18.5), (ii) normal weight (BMI between 18.5 and 24.9), (iii) overweight (BMI between 25 and 29.9), and (iv) obese (BMI ≥ 30).

Data on age, gender, education level, place of living, marital status, and smoking during the pandemic were collected. Socioeconomic status (SES) was evaluated based on scoring variables related to their household asset and wealth (homeownership, personal vehicle, washing machine, LCD/LED TV, dishwasher, laptop/computer, refrigerator, and microwave), education, and income. The SES score was computed for each respondent, and participants were then classified into three categories (low, medium, and high) using tertiles of the distribution of the SES scores as cutoff points.

In addition, respondents reported their health status, such as chronic diseases (diseases with proven diagnosis) and whether they were previously infected with COVID-19. According to the UK NHS report, if participants had any of 10 medical conditions (e.g., diabetes, weakened immune system, chronic kidney disease, etc.), they were considered to be “high risk” for COVID-19.

The present online survey was reported based on the Checklist for Reporting Results of Internet E-Surveys (CHERRIES) guidelines (19).

### 2.3 Ethics statement

The study protocol was approved by the ethics committee of Shiraz University of Medical Sciences (IR.SUMS.REC.1401.34).

### 2.4 Statistical analysis

The principal component analysis method was applied to identify dietary patterns. Dietary patterns were identified based on 18 food groups, eigenvalue (>1), factor interpretability, and the explained variance (>5%). Factors were rotated with varimax to achieve uncorrelated factors and improve the interpretability. Each food group received a factor loading associated with each dietary pattern. Factor loadings show the correlation coefficient between the food group and the dietary pattern. In the present study, food groups with factor loadings of more than 0.2 were considered to be important contributors to the pattern. After that, the factor score for each dietary pattern was computed by summing up intakes of food groups weighted by their factor loadings. Respondents received a factor score for each identified dietary pattern and were categorized into tertiles (three groups with equal sample size) of dietary patterns’ scores. Participants in the lowest tertile (T1) had the lowest adherence to the identified dietary pattern, and those in the highest tertile (T3) had the highest adherence to that dietary pattern.

The normality of the data distribution was evaluated by the one-sample Kolmogorov–Smirnov test. The quantitative variables were expressed as mean ± SD and qualitative variables as frequency (percentage). To compare the quantitative variables across tertiles of dietary patterns, a one-way analysis of variance (ANOVA) was used, while for categorical variables, the chi-square test was applied. To determine the association of dietary patterns with weight status and contracting COVID-19, multivariable logistic regression was applied. The odds ratios (ORs) and their 95% confidence intervals (95% CIs) were calculated in crude and adjusted models. In Model I, adjustment was made for the main confounders (age and gender). In Model II, additional adjustments were conducted for smoking status, marital status, and SES. The first tertile of dietary patterns was considered the reference category in all analyses.

## 3 Results

In total, 1,187 subjects were participated in the current study. Removal of participants who were not eligible (e.g., under 18 years) and participants who did not complete the survey resulted in a final sample size of *N* = 1,106.

Three major dietary patterns were identified using principal components analysis, and they were labeled as “Plant-based dietary pattern (DP),” “Meat DP,” and “Western DP.” These three dietary patterns explained 39.34% of the total variation in dietary intakes in this population. The “Plant-based DP” was characterized by high consumption of legumes, onions/garlic, fruits, nuts, dried fruits, vegetables, whole breads, and dairy. The “Meat DP” was mainly loaded with red meat, poultry, fish, and canned fish. The “Western DP” was associated with higher intakes of fast foods, carbonated drinks, fruit juice, soy, egg, and seeds. All food groups and their loading factors for each dietary pattern are presented in Table 1.

**Table 1:** Loadings of foods and food groups across major dietary patterns.

Food groups	Component		
	Plant-based dietary pattern	Meat dietary pattern	Western dietary pattern
Legumes	0.591	0.122	0.111
Onion/garlic	0.583		0.106
Fruits	0.581	0.273	0.213
Nuts	0.578	0.309	
Dried fruits	0.555	0.275	
Vegetables	0.539		0.203
Whole breads	0.462		−0.144
Dairy	0.344	0.318	
Red meat	0.227	0.776	
Poultry	0.128	0.752	0.149
Fish	0.128	0.723	
Canned fish		0.457	0.239
Carbonated drinks			0.650
Fast foods	−0.164	0.120	0.644
Fruits juice	0.164	0.275	0.600
Soy	0.325		0.504
Egg	0.257		0.416
Seeds	0.372		0.378

Extraction method: principal component analysis. Rotation method: varimax with kaiser normalization^a^.

The mean age and BMI of respondents were 34.5 ± 9.4 years and 26 ± 6.57 kg/m^2^, respectively; 85.2% of included subjects were female. The sociodemographic characteristics of participants across tertiles of the dietary patterns are presented in Table 2. The mean age of subjects was significantly higher in the upper tertile of plant-based DP compared to the first tertile (*p* = 0.01), while it was lower in the top tertile of meat DP and Western DP (*p* < 0.001). The percentage of women in the first tertile of Western DP was more than that in the third tertile. The frequency of participants who lived in urban areas was significantly higher in the third tertile of meat DP than in the first tertile (*p* < 0.001). Most subjects had bachelor’s and postgraduate degrees in the top tertile of plant-based DP and meat DP (*p* < 0.001), while a high frequency of the participants had bachelor’s and postgraduate degrees in the first tertile of Western DP (*p* < 0.001). The percentage of married subjects in the highest tertile of meat DP (*p* = 0.01) and Western DP (*p* < 0.001) were significantly lower than the first one. The proportion of respondents who had low SES in the first tertile of the plant-based DP and meat DP were notably higher compared to the top tertile (*p* < 0.001(, while it was higher in the third tertile of Western DP (*p* < 0.001). Furthermore, the percentage of participants who had a high risk for COVID-19 and had been diagnosed with COVID-19 was significantly lower in the last tertile of Western DP (*p* < 0.001). Body weight and BMI were not significantly different among the tertiles of the various types of dietary patterns.

**Table 2:** Sociodemographic characteristics of participants across tertiles of the dietary patterns.

Variables	Plant-based dietary pattern		*p-*value	Meat dietary pattern		*p-*value	Western dietary pattern		*p-*value
	Tertile 1	Tertile 3		Tertile 1	Tertile 3		Tertile 1	Tertile 3	
Sample size (*n*)	368	369		368	369		368	369	
Age (year)	33.37 ± 9.74	35.11 ± 9.67	0.01	35.22 ± 8.89	33.32 ± 10	<0.001	36 ± 9.2	32.14 ± 10	<0.001
Body weight (kg)	67.30 ± 13.42	69.24 ± 15.56	0.07	68.8 ± 14.9	68.32 ± 15	0.82	68.27 ± 14.79	68.49 ± 15.98	0.58
BMI (kg/m2)	25.72 ± 5.7	26.13 ± 5.8	0.49	26.27 ± 5.6	25.6 ± 5.48	0.30	25.94 ± 5.64	25.64 ± 5.86	0.61
Sex			0.76			0.31			<0.001
Male	54 (14.7)	51 (13.8)		46 (12.5)	60 (16.3)		37 (10.1)	76 (20.6)	
Female	314 (85.3)	318 (86.2)		322 (87.5)	309 (83.7)		331 (89.9)	293 (79.4)	
Place of living			0.76			<0.001			0.33
Urban	277 (75.3)	280 (75.9)		254 (69)	302 (81.8)		290 (78.8)	279 (75.6)	
Rural	91 (24.7)	89 (24.1)		114 (31)	67 (18.2)		78 (21.2)	90 (24.4)	
Education			<0.001			<0.001			<0.001
high school and less	80 (21.7)	50 (13.6)		83 (22.6)	45 (12.2)		47 (12.8)	78 (21.1)	
diploma	153 (41.6)	127 (34.4)		162 (44)	120 (32.5)		120 (32.6)	162 (43.9)	
Bachelor and Postgraduate	135 (36.7)	192 (52)		123 (33.4)	204 (55.3)		201 (54.6)	129 (35)	
Marital status			0.83			0.01			<0.001
Married (not separated)	270 (73.4)	290 (78.6)		300 (81.5)	270 (73.2)		291 (79.1)	262 (71)	
Widowed or divorced	7 (1.9)	7 (1.9)		8 (2.2)	3 (0.8)		4 (1.1)	9 (2.4)	
Single	91 (24.7)	72 (19.5)		60 (16.3)	96 (26)		73 (19.8)	98 (26.6)	
Smoking			0.37			0.83			<0.001
Non-smoker	344 (93.5)	351 (95.1)		347 (94.3)	346 (93.8)		358 (97.3)	328 (88.9)	
Smoker	24 (6.5)	18 (4.9)		21 (5.7)	23 (6.2)		10 (2.7)	41 (11.1)	
SES			<0.001			<0.001			<0.001
Low	260 (70.7)	197 (53.4)		283 (76.9)	173 (46.0)		199 (54.1)	253 (68.6)	
Moderate	100 (27.2)	152 (41.2)		81 (22)	161 (43.6)		38.9 (8.9)	105 (28.5)	
High	8 (2.2)	20 (5.4)		4 (1.1)	35 (9.5)		26 (7.1)	11 (3)	
At risk medical group for COVID			0.78			0.22			<0.001
Yes	116 (31.5)	112 (33.6)		133 (36.1)	112 (30.4)		127 (34.5)	99 (26.8)	
No	252 (33.9)	247 (33.2)		235 (63.9)	257 (69.6)		241 (65.5)	270 (73.2)	
Diagnosed COVID			0.44			0.41			<0.001
Yes	113 (30.7)	115 (31.2)		120 (32.6)	102 (27.6)		112 (30.4)	103 (27.9)	
No	368 (34)	369 (33.9)		255 (69.3)	254 (68.8)		256 (69.6)	266 (72.1)	

Quantitative variables: mean ± SD. Qualitative variables: frequency (percentage). *p* resulted from ANOVA for quantitative variables and the chi-square test for categorical variables.

### 3.1 Association of dietary patterns with BMI and contracting COVID-19

Multivariable-adjusted ORs (95% CI) for the association of dietary patterns with BMI and contracting COVID-19 are shown in Table 3. In the crude and adjusted models, no significant association was found between plant-based DP with underweight, obesity, and contracting COVID-19. In the case of the association of plant-based DP with overweight, the moderate adherence (second tertile) to plant-based DP was significantly related to increased odds of overweight (OR: 1.41; 95% CI: 1.02, 1.96); however, the relationship disappeared after adjusting for potential confounders. In the crude model, participants in the highest tertile of the Western DP had 2.62-fold higher odds (95% Cl: 1.32, 5.2) for underweight than the first one. However, this association disappeared after considering potential confounding variables.

**Table 3:** Multivariate adjusted odds ratio (OR) and 95% confidence interval (CI) for the association of dietary patterns with BMI and contracting COVID-19.

	Tertile 1	Tertile 2	Tertile 3	*P*-trend
**Plant-based dietary pattern**				
**Underweight**				
Crude model	1	0.78 (0.38, 1.5)	1.12 (0.58, 2.14)	0.73
Model 1	1	0.96 (0.46, 2.02)	1.30 (0.66, 2.54)	0.44
Model 2	1	1.02 (0.48, 2.14)	1.14 (0.71, 2.80)	0.32
**Overweight**				
Crude model	1	1.41 (1.02, 1.96)	1.24 (0.89, 1.72)	0.19
Model 1	1	1.29 (0.92, 1.82)	1.12 (0.80, 1.58)	0.49
Model 2	1	1.28 (0.90, 1.81)	`1.14 (0.80, 1.62)	0.45
**Obesity**				
Crude model	1	1.25 (0.82, 1.90)	1.19 (0.78, 1.82)	0.39
Model 1	1	1.14 (0.74, 1.76)	1.07 (0.69, 1.65)	0.73
Model 2	1	1.17 (0.75, 1.82)	1.16 (0.74, 1.81)	0.49
**Contracting COVID-19**				
Crude model	1	1.19 (0.88, 1.63)	1.02 (0.74, 1.39)	0.90
Model 1	1	1.14 (0.83, 1.55)	0.97 (0.71, 1.34)	0.89
Model 2	1	1 (0.79, 1.48)	0.92 (0.67, 1.28)	0.64
**Meat dietary pattern**				
**Underweight**				
Crude model	1	0.58 (0.28, 1.21)	0.95 (0.49, 1.82)	0.92
Model 1	1	0.57 (0.27, 1.20)	0.74 (0.37, 1.46)	0.42
Model 2	1	0.59 (0.28, 1.26)	0.80 (0.39, 1.62)	0.56
**Overweight**				
Crude model	1	0.78 (0.56, 1.09)	0.83 (0.59, 1.15)	0.27
Model 1	1	0.78 (0.56, 1.01)	0.93 (0.66, 1.33)	0.73
Model 2	1	0.82 (0.58, 1.17)	1.04 (0.72, 1.51)	0.79
**Obesity**				
Crude model	1	0.75 (0.50, 1.14)	0.68 (0.44, 1.03)	0.07
Model 1	1	0.76 (0.50, 1.17)	0.78 (0.50, 1.21)	0.28
Model 2	1	0.85 (0.55, 1.31)	0.99 (0.62, 1.58)	0.99
**Contracting COVID-19**				
Crude model	1	1.17 (0.86, 1.59)	0.79 (0.57, 1.08)	0.14
Model 1	1	1.16 (0.85, 1.58)	0.81 (0.58, 1.11)	0.20
Model 2	1	1.12 (0.82, 1.53)	0.73 (0.52, 1.02)	0.75
**Western dietary pattern**				
**Underweight**				
Crude model	1	1.03 (0.45, 2.35)	2.62 (1.32, 5.2)	<0.001
Model 1	1	1.05 (0.45.2.40)	1.83 (0.89, 3.76)	0.76
Model 2	1	1.02 (0.44, 2.37)	1.73 (0.83, 3.62)	0.11
**Overweight**				
Crude model	1	1.05 (0.76, 1.45)	0.99 (0.71, 1.37)	0.97
Model 1	1	1.10 (0.79, 1.54)	1.31 (0.92, 1.85)	0.12
Model 2	1	1.02 (0.72, 1.43)	1.25 (0.87, 1.79)	0.22
**Obesity**				
Crude model	1	1.79 (1.17, 2.74)	1.37 (0.88, 2.12)	0.17
Model 1	1	1.93 (1.24, 2.98)	1.96 (1.23, 3.13)	<0.001
Model 2	1	1.70 (1.09, 2.66)	1.75 (1.08, 2.81)	0.02
**Contracting COVID-19**				
Crude model	1	1.41 (1.04, 1.91)	0.88 (0.64, 1.21)	0.46
Model 1	1	1.41 (1.04, 1.92)	0.92 (0.66, 1.28)	0.70
Model 2	1	1.41 (1.03, 1.93)	0.94 (0.67, 1.32)	0.80

^1^Adjusted for age and gender. ^2^Adjusted for age, gender, smoking status, marital status, and SES.

Moreover, moderate adherence to Western DP was significantly associated with increased odds of obesity (OR: 1.79; 95% CI: 1.17, 2.74). This relation remained significant even after adjusting for confounders. In addition, high adherence to Western DP was significantly related to increased odds of obesity after controlling for confounders (model 1: OR: 1.96; 95% CI: 1.23, 3.13; model 2: OR: 1.75; 95% CI: 1.08, 2.81). Regarding contracting COVID-19, participants in the second tertile of the Western DP had 1.41-fold higher odds (95% Cl: 1.04, 1.92) for COVID-19 infection. This association remains significant after adjusting for confounding variables. However, neither crude nor adjusted models found any significant association among meat DP, BMI status, and contracting COVID-19.

## 4 Discussion

Our study aimed to investigate the association of major dietary patterns with socioeconomic status (SES), obesity, and contracting COVID-19 among Iranian adults. The findings from this cross-sectional survey provided valuable insights into the relationship between dietary patterns and these health outcomes. We found that participants with moderate and high adherence to Western DP had higher odds of obesity. In addition, we found that participants with moderate adherence to Western DP had higher odds of contracting COVID-19. However, we could not find any significant correlation between meat DP and plant-based DP with contracting COVID-19.

When examining the association between dietary patterns and BMI, we found that adherence to the Western DP was significantly associated with an increased likelihood of obesity. The Western DP is a widely followed eating pattern characterized by high salt, trans fats, saturated fats, and simple carbohydrates. However, it lacks complex carbohydrates and fibers. Moreover, it is considered a calorie-dense diet deficient in essential nutrients such as vitamins and minerals (20). These findings are consistent with previous studies that have shown the detrimental effects of Western DP on weight status. It has been reported in a population-based study that following the Western DP closely correlated with higher risks of obesity and weight gain (21). A cohort of healthy reproductive-age women study showed that adherence to the Western DP is associated with higher odds of obesity (OR: 2.68) (22). The findings of the cross-sectional study showed that modern dietary pattern (higher intake of soft drinks, fried foods, pickles, and lower intake of fresh vegetables) was positively associated with weight gain in men and women during the COVID-19 pandemic (23). In addition, our results align with a previous meta-analysis, indicating a 65% heightened risk of obesity linked to the Western DP (24). In terms of the mechanism, the Western DP led to hyperinsulinemia, which causes increased fat storage, appetite, hyperphagia, and carbohydrate craving (25).

Obesity is one of the main risk factors for increasing the risk of contracting COVID-19. Singh et al. (26) in a systematic review and meta-analysis study, showed that obesity was associated with an increased risk of severe disease and mortality rate among patients with COVID-19. In terms of odds of contracting COVID-19, we found that participants with moderate adherence to Western DP had significantly higher odds of COVID-19. In line with our findings, it has been reported in previous studies that moderate or higher adherence to Western DP led to a significant increase in the risk of infectious diseases such as COVID-19. Ebrahimzadeh et al. (17) in a cross-sectional study, showed that participants who followed unhealthy dietary patterns such as Western DP had a higher risk for COVID-19 and its related symptoms such as cough, fever, chilling, weakness, myalgia, nausea and vomiting, and sore throat.

Various mechanisms have been proposed that consumption of the Western diet increases the risk of chronic diseases such as fatty liver (27), cardiovascular diseases (28), metabolic syndrome (29), and infectious diseases. Long-term adherence to the Western dietary pattern increases the level of inflammatory factors (30), oxidative stress (31), and lipid peroxidation (32), all of which can be involved in the development of chronic diseases. In this study, unlike most of the previous studies, we considered the meat group as a separate dietary pattern, while in some studies, the high consumption of meats, especially red meat, is considered part of the Western dietary pattern.

Interestingly, the plant-based DP did not show a significant association with underweight, obese, or overweight and contracting COVID-19 in the adjusted models. In contrast to our findings, according to Kim et al.’s (33) population-based case–control study conducted in six countries, there was a significant 73% reduction in the likelihood of experiencing moderate-to-severe COVID-19 severity with a strong adherence to a plant-based DP. In addition, it has been reported in another study that higher adherence to plant-based DP caused a significant improvement against SARS-CoV-2 infection (16). Moreover, some studies indicated that higher adherence to the Mediterranean DP is negatively associated with COVID-19 infection-related deaths (34, 35).

Part of the contrast observed in our study with other studies could be due to the difference in the type of food selected in the plant-based DP and infection rates. Fruits and vegetables provide a wealth of vitamins, folate, fiber, and various phytochemicals such as carotenoids and flavonoids. In addition, whole grains are a rich source of group B vitamins, which have the ability to strengthen the endogenous antioxidant system (36–38). These compounds possess immune-protective properties due to their anti-inflammatory, antibacterial, and antiviral effects (39–41). It has been reported in a previous study that consuming a minimum of 0.67 daily servings of vegetables (excluding potatoes, whether cooked or raw) has been linked to a reduced risk of contracting COVID-19 (42). According to recent ecological studies on COVID-19, countries that have a significant intake of food rich in antioxidants or foods with anti-angiotensin-converting enzyme (ACE) activity, like raw or fermented cabbage, show a lower rate of COVID-19-related fatalities when compared to other countries (43, 44).

We could not find any significant correlation among meat DP, BMI status, and contracting COVID-19. However, Chen et al. (45) reported that students with a higher BMI consumed more meat products than others during the COVID-19 pandemic. Another study indicated that lower consumption of legumes, vegetables, and fruits and a higher intake of meat, sweets, salty snacks, and fast food are associated with obesity during the COVID-19 lockdown in Poland (46). In a population base study among the participants in the UK, it has been reported that participants in the 4th quartile of processed meat intake had higher odds for COVID-19, but in line with our findings, they did not find any significant correlation between total meat intake and COVID-19 (42). Sausages, bacon, and ham are the primary sources of processed meat consumption in many countries, and they frequently contain salt enriched with nitrates/nitrites (47). This type of meat also contains many preservatives and additives, many of which are harmful to health. Processed meats tend to have higher concentrations of both total and saturated fat (48, 49). Consumption of processed meats, commonly associated with a Western DP, might have a negative impact on immunity (50). Therefore, other dietary habits connected to processed meat intake may be responsible for the observed link with susceptibility to COVID-19.

This finding suggests that although plant-based DP is generally considered a healthy dietary pattern, moderate adherence to it may not have a significant impact on weight status among Iranian adults. Further research is needed to explore the potential factors influencing this result.

As a novelty, this study is the first one to evaluate the association of major dietary patterns with the risk of COVID-19 contracting. Our study has other strong points, including a large and diverse population sample. This evidence can be used to develop preventive measures against COVID-19 contracting and weight gain during the COVID-19 pandemic.

In interpreting the results of our study, it is important to acknowledge some limitations. First, the cross-sectional nature of the study design limits our ability to establish causality between dietary patterns and health outcomes. Second, the self-reported dietary assessment method might introduce recall bias and measurement error. Additionally, the reliance on self-reported weight and height for BMI calculation may introduce inaccuracies in the classification of weight status. Finally, the study sample mainly consisted of female participants, which may limit the generalizability of the findings to the entire Iranian adult population.

In conclusion, our study highlights the associations between major dietary patterns, socioeconomic status, obesity, and contracting COVID-19 among Iranian adults. The Western DP showed unfavorable associations with obesity and increased odds of contracting COVID-19, while the plant-based DP did not show significant associations with weight status or COVID-19 infection in the adjusted models. These findings emphasize the importance of promoting healthy dietary patterns, such as plant-based diets, for preventing obesity and potentially reducing the risk of COVID-19 infection. Future research should focus on longitudinal designs and more diverse populations to further investigate these associations.

## Data availability statement

The original contributions presented in the study are included in the article/supplementary material, further inquiries can be directed to the corresponding author.

## Ethics statement

Studies involving humans were approved by Research Ethics Committee of Shiraz University of Medical sciences (IR. SUMS.REC.1401.344). The current study was conducted in accordance with the local legislation and institutional requirements. Written informed consent for participation was provided by participants.

## Author contributions

MM: Conceptualization, Investigation, Methodology, Supervision, Writing – original draft. MR: Writing – original draft. AS: Writing – original draft, Investigation. SS: Data curation, Investigation, Methodology. SM: Conceptualization, Formal analysis, Methodology, Writing – original draft, Writing – review & editing.
